# 

**Published:** 1830-02

**Authors:** 


### MONTHLY LIST OF MEDICAL BOOKS.

[*Medical Works cannot be entered on this List except a copy be sent for the purpose; the titles of Books having frequently been transmitted to us, as published, which have not appeared for weeks, or even months, after.*]

Anatomy of the Brain and its Membranes, the Nerves, Organs of Sense, Arteries, Veins, and Lymphatics. Arranged in eight Tables. For the use of Students.—Burgess and Hill, 1830.

The anatomical student will find these tables very useful and convenient for reference.

A Letter to the Public on the Necessity of Anatomical Pursuits; with Reference to Popular Prejudices, and to the Principles on which Legislative Interference in these Matters ought to proceed. By Coaden Thompson, M.D.—8vo. pp. 92. J. Taylor, 1830.

Practical Remarks on Amputations, Fractures, and Strictures of the Urethra. By Stephen Love Hammick, Surgeon Extraordinary to the King, &c.—8vo. pp. 266. Longman, 1830.

A Treatise on Fever. By Southwood Smith, M.D. Physician to the London Fever Hospital.—8vo. pp. 436. Longman, 1830.

Clinical Illustrations of Fever; comprising a Report of the Cases treated at the London Fever Hospital, 1828, 1829. By Alexander Tweedie, M.D. Physician to the Fever Hospital, &c.—8vo. pp. 204. Whittaker and Co. 1830.

Medico-Chirurgical Transactions. Vol. XV. Part II. Plates.

A Synoptical, Meteorological, and Symptomatological Journal, or Medical Case-book of Record; containing Prescription Book and Ledger, thus affording also the most economical, accurate, easy, expeditious and efficient Mode of Medical Book-keeping hitherto invented, and indispensably requisite for all who are engaged in the Practice of Medicine and Surgery, in Hospitals, Dispensaries, or Private Practice, &c. By W. R. Russel Wilton, Surgeon, &c.—Longman, 1829.

The plan of this medical case-book is good; and if practitioners will take advantage of the facilities it offers to record the history and treatment of important cases, much benefit must accrue to the science of medicine in general.

A Synoptical Chart of Diseases of the Ear; showing their Order, Classification, Seat, Symptoms, Causes, and Treatment. By J. H. Curtis, Esq. Surgeon Aurist to his Majesty, &c.—Highley, London.

### METEOROLOGICAL JOURNAL,

### NOTICE TO CORRESPONDENTS.

Dr. H. *is mistaken in his conjecture.*

